# Noncoding RNAs: Emerging Players in Muscular Dystrophies

**DOI:** 10.1155/2014/503634

**Published:** 2014-03-04

**Authors:** Germana Falcone, Alessandra Perfetti, Beatrice Cardinali, Fabio Martelli

**Affiliations:** ^1^Institute of Cell Biology and Neurobiology, National Research Council, 00015 Monterotondo Scalo, Italy; ^2^Policlinico San Donato-IRCCS, Molecular Cardiology Laboratory, 20097 San Donato Milanese, Milan, Italy

## Abstract

The fascinating world of noncoding RNAs has recently come to light, thanks to the development of powerful sequencing technologies, revealing a variety of RNA molecules playing important regulatory functions in most, if not all, cellular processes. Many noncoding RNAs have been implicated in regulatory networks that are determinant for skeletal muscle differentiation and disease. In this review, we outline the noncoding RNAs involved in physiological mechanisms of myogenesis and those that appear dysregulated in muscle dystrophies, also discussing their potential use as disease biomarkers and therapeutic targets.

## 1. Introduction

In the past decade noncoding RNAs (ncRNAs) and their physiological and pathological functions have been the focus of intense research interest. These RNAs constitute the majority of the transcriptome and are never translated into proteins. In addition to the better known “house-keeping” ribosomal RNAs (rRNAs), transfer RNAs (tRNAs), small nuclear RNAs (snRNAs), and small nucleolar RNAs (snoRNAs), the remaining ncRNAs have been recently established as key regulators of gene expression in virtually all biological processes. In particular, two classes of ncRNA molecules with regulatory functions have attracted much attention: microRNAs (miRNAs) and long noncoding RNAs (lncRNAs). miRNAs act posttranscriptionally to repress the function of target mRNAs. lncRNAs, more than 200 nucleotides long, are localized either in the nucleus, where they can be associated with chromatin-remodeling complexes to regulate transcription, or in the cytoplasm, acting as posttranscriptional regulators. In this review, the emerging role of these ncRNAs in muscular dystrophies will be discussed.

## 2. Noncoding RNAs

### 2.1. miRNAs

**Figure 1:**
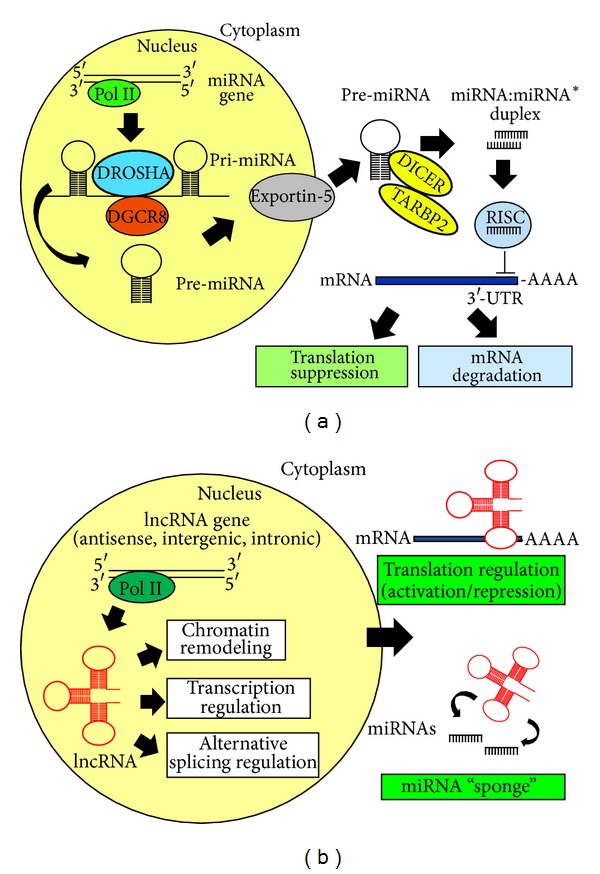
Biogenesis and mechanisms of action of miRNAs (a) and lncRNAs (b).

miRNAs are short (19–24 nt), single-stranded ncRNAs that regulate gene expression at the posttranscriptional level, either by cleavage of target mRNAs or by repressing their translation [1, 2]. miRNAs likely contribute to the regulation of most biological functions, as more than half of the human transcriptome is predicted to be under their regulation [3, 4]. miRNA biogenesis and maturation is a complex multistep process. miRNA genes are generally transcribed by RNA polymerase II either as part of introns of mRNA genes or from intergenic regions. Interestingly, multiple miRNAs can be excised from a single, multicistronic, pri-miRNA transcript that can include multiple members of a miRNA family, as well as unrelated miRNAs. The primary transcripts (pri-miRNAs) are then cleaved in the nucleus by the DROSHA-DGCR8 microprocessor to generate approximately 70-nt long hairpin-shaped precursors called pre-miRNAs [5]. The transport of pre-miRNAs from the nucleus to the cytoplasm is mediated by exportin-5, a RanGTP-binding nuclear transporter [6, 7]. In the cytoplasm, the RNAse III-like enzyme DICER and TARBP2 (TAR binding protein 2) cleave the pre-miRNA into a transient duplex of around 20–24 nt in size made up of the functional miRNA strand and the passenger strand [6, 7]. The mature miRNA binds to Argonaute (Ago) proteins to form a miRNA-induced silencing complex termed RISC, which mediates gene silencing by mRNA degradation or translation inhibition [8, 9]. Target recognition by miRNA depends on base pairing between miRNA seed sequence (nt 2–8 at the 5′ end) and sequences usually located in the 3′ UTR of the target mRNA (Figure 1(a)). The outcome of gene silencing, either mRNA degradation or translation inhibition, appears to be determined by degree and nature of the complementarity between the miRNA and the target mRNA [4, 10–12]. Interestingly, it has been recently shown that translational inhibition precedes mRNA degradation and is necessary for mRNA degradation by miRNAs [13]. A single miRNA can inhibit several targets and a single mRNA can be targeted by multiple miRNAs in a combinatorial way [14]. In addition, families of miRNAs comprise members with identical seed sequences and are thought to share the same targets; this redundancy may be necessary to reinforce and stabilize regulation of important pathways.

### 2.2. lncRNAs

The advent of full genome sequencing techniques led to the discovery that the genome encodes at least as many lncRNAs as the known protein-coding genes. lncRNAs are a very heterogeneous group of RNA molecules, both in structure and function. They have been tentatively classified on the basis of their position with respect to protein coding genes as antisense lncRNAs, intronic lncRNAs, and long intergenic noncoding RNAs (lincRNAs) [15]. Similar to protein-coding mRNAs, they can be spliced from multiexonic precursors, have a 5′-cap, and be polyadenylated; many nonpoliadenylated lncRNAs have also been identified [16]. Recent studies revealed that lncRNAs tend to share some properties such as a tendency for location next to developmental regulators, an enrichment of tissue-specific expression patterns, and a certain degree of evolutionary conservation in functional domain-containing sequences and predicted secondary structure [17–19]. In the recent years, thanks to the availability of new powerful technologies, novel lncRNAs have been discovered, bringing the total number of human lincRNAs to many thousands [20]. The molecular mechanisms by which lncRNAs exert their function are poorly understood, and only for a limited number of them a function has been defined that implicates their involvement in numerous cellular processes ranging from embryonic stem cell pluripotency, cell-cycle regulation, and diseases. Intracellular localization is often used as a predictive element to get insights into lncRNA molecular mechanisms [21]. Nuclear lncRNAs can act as a decoy for splicing factors [22, 23] as well as both *cis*- and *trans*-regulators of gene activity and modulators of the epigenome [15, 24]. Among them, particularly interesting is a class of lncRNAs transcribed from regulatory elements and proposed to take part in the gene regulatory networks [25]. These transcripts, called eRNAs, are described as a rare population of 0.5–5 kb transcripts, some of which undergo polyadenylation. Recent data suggest that eRNAs contribute to establish a cell-type-specific transcriptional circuitry by directing chromatin-remodeling events at specific loci, including the MYOD1 locus [26]. Finally, cytoplasmic lncRNAs may function as endogenous “sponges” for miRNAs, thus releasing miRNA repression on target genes [27, 28] (Figure 1(b)).

## 3. Myogenesis and ncRNAs

During embryonic development the integration of numerous synergistic signaling pathways turns a single cell into a multicellular organism with specialized cell types and highly structured, organized tissues. Vertebrate trunk skeletal muscle derives from the somites that were progressively subdivided into embryonic compartments giving rise to dermomyotome and subsequently to myotome to produce differentiated muscular tissue [45]. After initial proliferation, myoblasts withdrew from the cell cycle, accumulated muscle-specific proteins, fused into multinucleated myotubes, and assembled specialized contractile structures. Skeletal myogenesis is coordinated by the activation of the myogenic regulatory factors (MRFs) in response to the upstream regulators paired domain- and homeobox-containing proteins Pax3 and Pax7 expressed in different precursor cells during development. The MRFs then trigger a cascade of transcription factors and downstream structural genes, ultimately resulting in the generation of the specific histotypes. Analyses of embryos carrying null mutations of the MRFs, either singly or in combination, have led to the view that Myf5 and Mrf4 operate at the top of the myogenic cascade, MyoD operates downstream of them in some precursors, in parallel in others, and myogenin acts as the final effector, controlling terminal differentiation [46].

**Figure 2:**
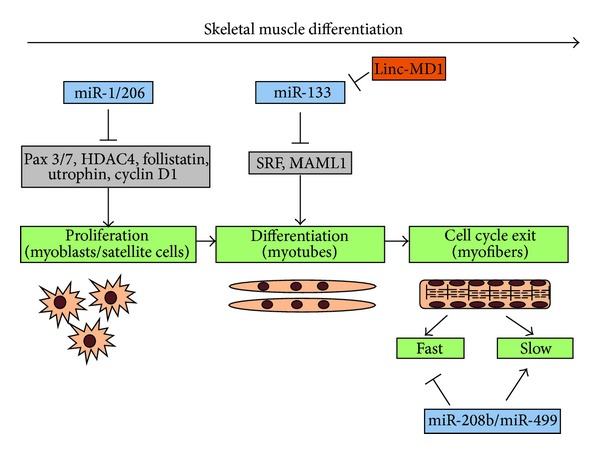
Regulation of the myogenic differentiation program by myomiRs and lncRNAs.

**Figure 3:**
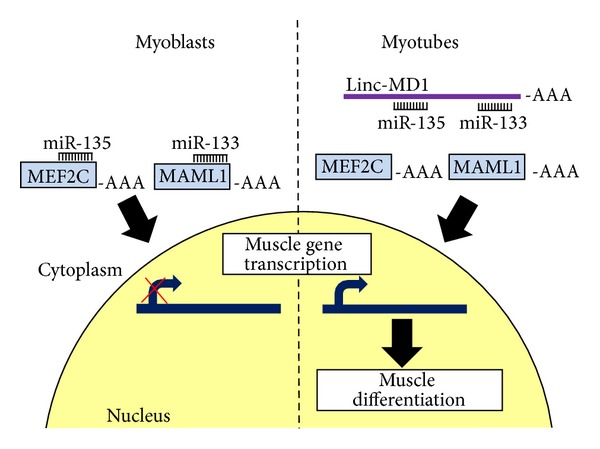
Linc-MD1 regulation of myogenic differentiation.

It is now well established that key aspects of skeletal muscle biology are subject to regulation by miRNAs. The importance of miRNAs in skeletal muscle development is demonstrated by the fact that the muscle-specific knockout of Dicer in mice results in decreased skeletal muscle mass accompanied by abnormal myofiber morphology and perinatal death [47]. One particular group of miRNAs, the myomiRs (miR-1, miR-206, and miR-133a/b), is highly and specifically expressed during cardiac and skeletal muscle cell differentiation, with miR-206 being the only myomiR specific to skeletal muscle [48, 49]. Notably, their functions are conserved from vertebrates to invertebrate species including *Drosophila* [50] and *C. elegans* [51]. MyomiRs are expressed during somite myogenesis and during muscle differentiation in cell culture models [52–55] and in developing embryos [49, 56, 57]. Indeed, the expression of these miRNAs is directly regulated by MRFs [58, 59]. Although these miRNAs are closely linked and share at least some regulatory elements, they have been shown to exert opposite effects in muscle differentiation, possibly mediated by distinct mRNA targets. When overexpressed in cultured myoblasts, miR-1/206 promotes differentiation through inhibition of histone deacetylase 4 (HDAC4), whereas miR-133 promotes proliferation possibly through inhibition of serum response factor [53]. miR1/206 targets in myoblasts include follistatin, utrophin, and cyclin D1 [55, 60] and in satellite cells include Pax3 and Pax7; all factors required to maintain cell proliferation [61–63] (Figure 2). miR-1 has been shown to be also induced by IGF-1 (insulin-like growth factor 1), a well-known regulator of muscle growth and development. In turn, IGF-1 and its receptor are both predicted as targets of miR-1, thus generating a feedback loop between the IGF signaling pathway and miR-1 expression in muscle differentiation [64]. Recently, a cross-regulation between miR-133 and a muscle-specific lncRNA, named MD1, has been described (Figure 2). MD1 is a lincRNA that generates miR-133 and is itself a miR-133 target, since it contains miR-133, as well as miR-135, target sequences. Through these sequences, during differentiation, MD1 can sequester miR-133 and miR-135 competing for binding to their normal targets, thus acting as a natural decoy for the two miRNAs [28]. In particular, two targets of miR-133 and miR-135, MAML1 and MEF2C, respectively, both are positive regulators of myogenic differentiation, which have been shown to be upregulated following MD1 induction upon differentiation [28] (Figure 3).

Two additional muscle-specific miRNAs are miR-208b/miR-499 that are generated from the introns of two myosin genes, *β*-*MHC* and *Myh7b*. They are functionally redundant and play a dominant role in the specification of muscle fiber identity by activating slow and repressing fast myofiber gene programs [65].

Many nonmuscle-specific miRNAs also play key roles in regulation of myogenesis. In addition to miR-1/206, also miR-27 and miR-486 target Pax3 and Pax7, respectively [63, 66]. The TGF-*β* signaling pathway, a negative regulator of myogenic differentiation, is subjected to a complex miRNA regulation, while miR-26a promotes differentiation by targeting the transcription factors Smad1 and Smad4, critical for the TGF-*β* pathway [67]; TGF-*β* signaling controls myogenesis through downregulation of miR-24 [68] and of miR-206 and miR-29 via altered regulation of HDAC4 [69]. Nonmuscle-restricted miR-221 and miR-222, which target the cell-cycle regulator p27, are downregulated during muscle cell differentiation and, when overexpressed, can delay cell-cycle withdrawal and inhibit myocyte fusion and myotube maturation [70]. Another regulator of myogenesis is miR-125b, which targets IGF-2, an important regulator of muscle cell growth [71]. miRNAs promoting myogenic differentiation include miR-181, which regulates HOXA11 (homeobox A11) during muscle differentiation [72], miR-378 which downregulates MyoR, a repressor of myogenic differentiation that antagonizes MyoD [73], and miR-214, which targets both EZH2 (enhancer of zeste homologue 2), part of the polycomb complex controlling epigenetic modifications of chromatin [74] and N-ras, the downregulation which facilitates cell-cycle exit [75]. In addition, miR-29 targets Akt3 to reduce proliferation and facilitate differentiation of skeletal myoblasts [76], and miR-199 suppresses the WNT-signaling factors FZD4, JAG1, and WNT2 which act to balance myogenic cell proliferation and differentiation [29].

Taken together, these findings clearly highlight the role of ncRNAs as crucial regulators of the myogenic differentiation program.

## 4. Muscular Dystrophies

Changes in the physiological demands of skeletal muscles induce responses that can involve modifications in the overall mass of the tissue, the spatial relationships among muscle cells, and components of the extracellular matrix, or the reprogramming of gene expression to alter specialized metabolic and contractile properties. As for physiological adaptations, pathological conditions also provoke remodeling responses in muscle tissue, which initially lead to impaired contractile performance and ultimately in clinical deterioration. In myopathies one of the most severe features is the progressive loss of skeletal muscle tissue due to chronic degeneration. Albeit generally at later stages, the heart is often involved. The muscular dystrophies are a heterogeneous group of over 30 different inherited disorders all involving progressive weakness and degeneration of skeletal muscle with variable distribution and severity, resulting in significant morbidity and disability, manifesting at any age from birth to senescence [77]. The diseases are defined and classified according to their genetic cause as well as clinical and pathological manifestation, the distribution of predominant muscle weakness, and the involvement of other organs [77]. A comprehensive survey of ncRNAs in all types of muscular dystrophies and myopathies is beyond the scope of this review. Here, the most relevant findings on the role played by ncRNAs in the most common muscular dystrophies, Duchenne muscular dystrophy (DMD), Becker muscular dystrophy (BMD), myotonic dystrophy (DM), and facioscapulohumeral muscular dystrophy (FSHD) will be discussed.

### 4.1. DMD and BMD

DMD is a severely debilitating neuromuscular disorder affecting 1 in 3,500 males. It is manifested by rapidly progressive proximal muscle wasting starting around 3 years of age, culminating with respiratory insufficiency and cardiac failure that leads to premature death by the mid 20s. The allelic disorder BMD is less common and milder, with late disease onset and relatively advanced survival age. Both diseases are caused by mutations in the *DMD* gene, the largest gene in the human genome, located on the X chromosome, which encodes the 427-kD protein dystrophin [78–81]. DMD is caused by recessive, frameshifting deletions and duplications or nonsense mutations that lead to complete loss or expression of nonfunctional dystrophin in myofibers, whereas mutations causing BMD produce semifunctional dystrophin [78, 82, 83].

Comprehensive miRNA expression profiling has revealed that miRNA dysregulation is a common feature of muscle pathology. Eisenberg et al. [30] described a miRNA expression profile in muscle tissues from several human primary muscle disorders and identified a series of miRNAs that are regulated either in almost all myopathies analyzed or specifically in DMD. In addition, a strong functional correlation was observed in DMD between downregulated mRNAs and predicted miRNA targets, suggesting a tight posttranscriptional regulation at the mRNA level in this disease. Indeed, miR-206 expression was significantly increased in the diaphragm and in regenerating and newly formed fibers of *mdx* mice, a well-established mouse model of DMD [31, 32], and in DMD patient biopsies [30, 33]. One particularly muscle-enriched miRNA, miR-486, was significantly downregulated in dystrophin-deficient mouse and human skeletal muscles. Interestingly, miR-486 levels were not reduced in biopsies of BMD patients, where a partially functional dystrophin protein is present [30]. Inhibition of miR-486 in normal muscle myoblasts resulted in reduced cell migration and wound repair, whereas its overexpression resulted in increased proliferation [34]. Transgenic mice overexpressing miR-486 exhibit impaired muscle regeneration and altered expression levels of PTEN/Akt signaling components [34]. miR199a-5p was previously found modulated in various muscle diseases [30] and upregulated in dystrophic zebrafish, mice, and human muscle [29]. This miRNA was also shown to be a regulator of myogenic progression in normal and dystrophic muscle by potentially modulating the expression levels of WNT signaling components [29].

Another study reported a correlation between miRNA expression profiles in DMD patient tissues and in *mdx* mice, both lacking a functional dystrophin gene [33]. Eleven miRNAs were deregulated both in *mdx* mice and in DMD patients. According to their expression, DMD-specific miRNAs were divided into 3 classes: regeneration-associated miRNAs (miR-31, miR-34c, miR-206, miR-335, miR-449, and miR-494), which were induced in *mdx* mice and in DMD patients and three of which (miR-206, miR-34c, and miR-335) were upregulated following myoblast differentiation *in vitro*; degenerative-miRNAs (miR-1, miR-29c, and miR-135a) that were downmodulated in *mdx* mice and in DMD patients and linked to myofiber loss and fibrosis; inflammatory miRNAs (miR-222 and miR-223) which were expressed in damaged muscle areas and whose expression correlated with the presence of infiltrating inflammatory cells. Besides the hypothesized role in inflammatory response, miR-222 could play a specific role in muscle fiber regulation, since its overexpression in cultured myocytes results in defective fusion and myotube morphology [70]. In agreement, beta1-syntrophin, a component of the dystrophin-glycoprotein complex (DAPC) that is altered in DMD and BMD, was shown to be a target of miR-222, and its expression was found downregulated in mouse dystrophic muscles where miR-222 levels are increased [35]. Dystrophin has been reported to be a direct target of miR-31 in cultured myoblasts and miR-31 expression is increased in human DMD samples; accordingly, human DMD myoblasts, undergoing an exon skipping inducing treatment, showed rescue of dystrophin expression following miR-31 inhibition [36]. Although some miRNAs have been shown to exert a disruptive effect on skeletal muscle differentiation at least in cell culture models [70], in most cases, it remains to be established whether the changes in miRNA expression levels are causally involved in these diseases or are secondary to the degeneration/regeneration response of the affected muscle tissue. Manipulation of miRNA levels in dystrophic mouse models will help clarify this issue.

An interesting correlation was found between DMD pathology and downregulation of linc-MD1 [28]. Compared with control cells, DMD patients-derived myoblasts showed a reduced ability to undergo terminal differentiation, accompanied by a reduced and delayed accumulation of muscle-specific markers such as myogenin and MHC. In DMD myoblasts levels of linc-MD1 were severely reduced and this, together with the unrestricted accumulation of miR-135, likely determined low levels of its target MEF2C; conversely, the strong downregulation of miR-133 correlated with the upregulation of MAML1 (Figure 3). Similar results were also obtained during differentiation of satellite cells derived from wild-type and *mdx* mice [28]. These data, besides reinforcing the importance of linc-MD1 as positive regulator of skeletal muscle differentiation, highlight the relevance of its downregulation in the pathogenesis of DMD [84].

A list of most relevant ncRNAs dysregulated in dystrophic skeletal muscle is shown in Table 1.

### 4.2. DM

DM is the most common form of muscular dystrophy in the adult. The disease is chronic and slowly progressing, with symptoms that include loss of muscle strength, myotonia, and excessive fatigue, with variable degree of severity. Although muscular dystrophy is the most prominent feature of the pathology, DM is a multisystemic disease and many patients present with cardiac arrhythmias, cataracts, insulin resistance, cognitive impairment, and serological alterations [85]. There are two forms of DM, named DM1 and 2; the first and more common one is caused by an expanded (CTG)*n*, in the 3′ untranslated region of the dystrophia myotonica protein kinase (*DMPK*) gene and the second consists in the expansion of (CCTG)*n* in the first intron of the *CNBP *(cellular nucleic acid binding protein) gene, previously named *ZNF9*. Phenotypes of DM1 and DM2 are similar but there are some important differences, most conspicuously in the severity of the disease, muscles primarily affected, involved muscle fiber types, and some associated multisystemic phenotypes [85]. The pathogenic mechanism of DM1 and DM2 is thought to be mediated by the mutant RNA transcripts containing expanded CUG and CCUG repeats that have been associated with a toxic RNA gain of function. Expanded repeats have been demonstrated to be toxic *per se* in several cell types and animal models [86–88], disrupting transcription and alternative splicing of several genes and pre-mRNAs in mice [89]. Expanded CUG repeats sequester nuclear proteins and accumulate into distinctive foci within muscle and neuronal nuclei [90]. The splicing factor Muscleblind-like 1 (MBNL1) is recruited into these foci, causing loss of function of the protein, which has been linked to critical DM1 features [89, 91]. The newly discovered function of the MBNL1 protein as a cytoplasmic regulator of miRNA biogenesis implicates an alteration of the miRNA processing pathway in the RNA toxicity that occurs in DM1 [37]. The study by Rau et al. [37] demonstrated that the altered processing of miR-1, consequent to insufficient availability of MBNL1, is linked to heart defects in DM1 patients and also provided a mechanistic explanation for this observation. As many other miRNA precursors contain sequence motifs recognized by MBNL1 in their hairpin loops, the search for further miRNAs deregulated by the same mechanism in DM1 tissue may provide insight into the scale of miRNA deregulation. Moreover, nuclear and/or cytoplasmic step of miRNA processing could be affected by the sequestration of important RNA binding proteins by the expanded repeats, thus contributing to RNA toxicity.

miRNA profiling in DM1 muscle biopsies revealed that miR-206, miR-1, and miR-335 are overexpressed, whereas miR-29b, miR-29c, and miR-33 are downregulated. However, independent studies have found neither changes in miR-1 levels nor a reduction of this miRNA, due to defective maturation of the precursor [37–40]. In addition to miR-1, also miR-7 and miR-10 were found downregulated in a DM1 *Drosophila* model as well as in DM1 patient-derived cells [40] (Table 1). Importantly, the intracellular localization of myomiRs miR-1, miR-133b, and miR-206 was severely altered, and notably, in spite of miR-1 upregulation, expression levels of its predicted targets were also found increased, possibly due to altered miR-1 function [39]. This highlights that miRNA level measurement alone is insufficient to define a miRNA involvement in pathogenetic mechanisms, but an accurate analysis of its intracellular distribution and target association is required. Searching for “functional” miRNAs in muscle biopsies, actually RISC-associated and engaged in mRNA target downregulation, may help solving these discrepancies and addressing unresolved issues.

In a recent study, miRNA expression levels were measured by qPCR array analysis in the skeletal muscle of DM2 patients, leading to identification of a subset of miRNAs that are specifically deregulated, potentially contributing to DM2 pathogenetic mechanisms. Nine miRNAs were found upregulated and four downregulated compared to controls. Interestingly, some of them (miR-193b-3p, miR-208a, and miR-381) were similarly modulated in skeletal muscle of DM1 patients [41] (Table 1).

Parallel to miRNA deregulation, the involvement of the RNA interference (RNAi) pathway in DM pathogenesis has been proposed. Trinucleotide repeated transcripts derived from mutated genes can form double-stranded RNAs, either as a result of bidirectional transcription or simply for their secondary structure. These transcripts can be cleaved by the ribonuclease Dicer into 21 nucleotides CAG/CUG repeat RNAs potentially active in silencing through an Ago-2-dependent manner. In a DM1 cell model it was demonstrated that transcripts containing long CUG and CAG repeat hairpins are substrates of Dicer and that fragments of the repeat sequences produced by Dicer act as endogenous siRNAs and trigger the downstream silencing effect [92]. More recently, the RNAi mechanism was described in human cell lines expressing mutant CAG repeats in the sequence context of the Huntingtin gene, causally involved in Huntington's disease. Mutant CAG repeats gave rise to toxic small RNA (sCAG) in a Dicer-dependent manner and caused a downstream silencing effect in an Ago2-dependent manner [93]. Taken together, both the miRNA and RNAi pathways appear to contribute to the RNA toxicity triggered by expanded CAG and CUG repeats, but the relevance of this contribution to pathology remains to be determined.

### 4.3. FSHD

FSHD is a neuromuscular disorder often considered to be the third most common muscular dystrophy characterized by progressive wasting of facial, upper arm, and shoulder girdle muscles. The disease is not caused by classical mutations in a protein-coding gene, but it correlates with reduction in the copy number of the 3.3 kb macrosatellite D4Z4 repeat mapping in the subtelomeric region of human chromosome 4 long arm (4q35). These deletions are associated with disruption of chromatin architecture by unknown mechanisms, highlighting that important epigenetic components are involved in the genesis of FSHD. A possible involvement of miRNAs in FSHD has been suggested [94]. Sense and antisense transcripts originating from the D4Z4 region have been identified, which might generate double-stranded RNA subsequently cleaved by Dicer to generate small siRNA/miRNA-sized fragments. The transcripts and small RNA fragments identified at the D4Z4 repeats might be associated with local chromatin silencing, chromatin silencing at distant loci, or might target RNA from other loci. However, further studies are needed to confirm whether or not these small RNAs are functional miRNAs [94]. A recent study described a simultaneous miRNome/transcriptome analysis in primary myoblasts from healthy subjects and FSHD patients where 29 miRNAs were found differentially expressed in FSHD samples [42] (Table 1). Twelve of these miRNAs, including miR-1, miR-206, miR-133a, and miR-133b myomiRs, were induced by overexpression of DUX4c transcription factor, encoded within the D4Z4 DNA region. Despite upregulation of several myogenic microRNAs, premature myogenic differentiation of FSHD myoblasts was not observed and, notably, this correlated with lack of suppression of some of their targets [42].

Interestingly, a chromatin-associated noncoding RNA, DBE-T, has been recently identified which is produced selectively in FSHD patients and coordinates derepression of 4q35 genes. Cabianca et al. [95] showed that the Polycomb group of epigenetic repressors targets D4Z4 in healthy subjects and that D4Z4 deletion is associated with reduced Polycomb silencing in FSHD patients. DBE-T recruits the Trithorax group protein Ash1L to the FSHD locus, driving histone H3 lysine 36 dimethylation, chromatin remodeling, and 4q35 gene transcription. The activation by DBE-T of certain genes normally repressed by the Polycomb complex, such as the transcription factor coding gene DUX4, results in significant cell toxicity and in downregulation of MyoD, contributing to the FHSD phenotype [96, 97].

## 5. ncRNAs in Therapeutic Perspectives

While the genetic mutations causing most muscular dystrophies have been identified, allowing a careful and unambiguous diagnosis by genetic tests, therapeutic intervention is mainly directed to relieve secondary symptomatic effects rather than targeting the primary causes of the diseases.

In DMD, corticosteroids have been shown to improve skeletal muscle strength and function in reproducible randomized controlled trials [98–100], but their efficacy lasts only for a few years, and adverse effects often result in discontinuation of treatment [101]. Other pharmacologic therapies are primarily directed toward managing comorbidities (such as cardiomyopathy, osteoporosis, and respiratory failure) [102].

Likewise, a valid therapy is not yet available for DM, and only symptomatic treatment is administered. For example, mexiletine, a local anesthetic and class 1B antiarrhythmic drug, reduces handgrip relaxation time in DM1 [103, 104], cardiac arrhythmia is often treated with a pacemaker or an implantable cardioverter defibrillator [105], cataracts require conventional surgery, and hypothyroidism and gonadal failure are treated with hormone replacement [85].

Similarly, no disease-specific therapeutic strategies are available for FSHD at the present time. For the muscle pain, the use of nonsteroidal anti-inflammatory drugs is warranted in patients with FSHD, while physiotherapy improves patients' functional status [106].

The development of new therapeutic tools directed to modulate disease effector molecules is therefore required.

### 5.1. miRNAs as Biomarkers

In addition to their implication in disease mechanisms, miRNAs are also attractive potential biomarkers. The serum protein creatine kinase released from damaged fibers, routinely measured to monitor skeletal muscle pathologies, is a useful biomarker, which is however not specific for a given pathology and, compared to miRNAs, correlates poorly with the severity of the disease [36]. Circulating miRNAs represent ideal biomarkers, since they are stably maintained into the extracellular environment and can be analyzed and quantified by relatively simple, fast, and inexpensive methods. It has been shown in human and in animal models that the circulating miRNA expression profile is dynamically changing in correlation with the pathophysiological state of the affected subjects and, in multisystemic pathologies such as muscular dystrophies, is representative of the different affected tissues, integrating their tissue-specific effects [107, 108]. Specifically, increased serum levels of miR-1, miR-133, and miR-206 have been detected in mice and dog models of DMD as well as in DMD patients compared to normal controls. In *mdx* mice, serum levels of these miRNA are lowered in animals following exon skipping inducing treatment [36, 43], suggesting that their levels correlate with disease severity. Interestingly, unlike creatine kinase levels, a biomarker for muscular diseases including DMD and expression levels of these miRNAs in *mdx* serum were little influenced by physical exercise [43].

In a recent study, serum miRNA levels in four mouse models of muscular dystrophy and one of hypertrophic cardiomyopathy (HCM) were determined: in DMD, limb-girdle muscular dystrophy type 2D and limb-girdle muscular dystrophy type 2C mouse models, which all exhibit massive myofiber destruction, very similar miRNA alterations were detected, confirming miR-1, miR-133, and miR-206 as the most deregulated species, whereas in Emery-Dreifuss mice, where massive muscle damage is uncommon, these miRNAs were down- rather than upregulated [44]. The dysregulated miRNAs identified in the HCM model were different, with the exception of one miRNA common to all disease models, miR-200a. Transferability of these results to humans, however, requires further investigation. Indeed, when a small DMD patient group was assayed, expression levels of only 5 out of 9 miRNAs were found similarly altered [44].

A list of circulating miRNAs found dysregulated in muscular dystrophies is shown in Table 2.

Identification of disease-specific circulating miRNA profiles could be employed for diagnosis and monitoring the outcomes of therapies. However, a larger collection of data from patients is required to establish a precise correlation between miRNA levels and disease.

### 5.2. ncRNAs as Therapeutic Targets

#### 5.2.1. miRNAs

**Figure 4:**
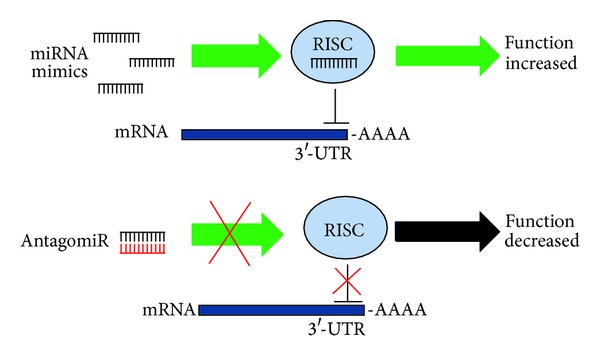
Therapeutic strategies targeting miRNAs.

As previously shown, several miRNAs are significantly dysregulated in muscular dystrophies. The ability to inhibit miRNA function through the use of complementary sequences makes miRNAs an attractive candidate for therapeutic treatments, also considering that a single miRNA or miRNA family can regulate many target genes and influence a whole gene network [30]. Several molecular tools have been developed to reduce the levels of pathogenic or aberrantly expressed miRNAs such as locked nucleic acids (LNAs) or antagomirs (cholesterol-modified) single stranded nucleic acids consisting of the complementary miRNA sequence (Figure 4). These inhibitors have been shown to effectively decrease miRNA levels in cell culture and animal models (reviewed in [109]). The feasibility of miRNAs as therapeutic targets in human pathologies is confirmed by a recent report describing the successful use of the LNA inhibitor of miR-122, called miravirsen, to treat hepatitis C virus infection in a phase 2a study [110]. Importantly, the drug was very well tolerated, encouraging the further use of this new class of LNA drugs. This latter aspect is very important since innate immune response to synthetic RNA has been reported [111].

A modest number of miRNAs, both skeletal muscle-specific and nonskeletal muscle-specific, are significantly downregulated in various myopathies [30, 33, 39]. With an approach reciprocal to miRNA inhibition, the use of miRNA mimics represents an attractive tool to boost the expression of downregulated miRNAs (Figure 4). Although comparatively fewer studies adopting this strategy are present in the literature, a clinical trial is now underway employing MRX34, a liposome-formulated mimic of the tumor suppressor miR-34a (http://clinicaltrials.gov/show/NCT01829971/). The aim of this phase 1 study is to evaluate the safety of MRX34 in patients with primary liver cancer or those with liver metastasis from other cancers.

Another potential therapeutic approach is represented by the use of viral vectors for the delivery of either miRNA mimics or decoys [112]. Vectors encoding transcripts containing multiple tandem-binding sites to a specific miRNA, known as miRNA sponges, have been developed, which can effectively titer away the aberrantly expressed miRNA species from its endogenous targets and can be potentially used to inhibit one or more miRNAs [113]. While these approaches have been successfully used in animal models, safety and efficiency of delivery concerns apply as for any other gene therapy protocol employing viral vectors in humans (reviewed in [114]). In addition, specificity and selectivity in targeting miRNAs in the affected tissue is an important requirement especially if nonskeletal muscle-specific miRNAs are to be used as a treatment for myopathies.

#### 5.2.2. lncRNAs

The pervasiveness of lncRNAs in human disease is now beginning to be understood. Thus, compared to other RNAs, therapeutic targeting of lncRNAs has been poorly investigated so far. Theoretically, experimental strategies of lncRNA manipulation for therapeutic purpose display all the opportunities and difficulties of mRNA targeting, but a deeper knowledge of the specificities of this class of RNA is needed [115–118]. As an example, RNAi could be used to target a repressor lncRNAs and this would result in de-repression of the lncRNA-regulated gene and activation of gene expression [119].

A particular challenge is represented by nuclear lncRNAs that are not accessible, in most circumstances, by the cytoplasmic RNA silencing machinery [119, 120]. In this respect, a similar issue is posed by the nuclear accumulation of transcripts containing CUG expansion in DM1. Strategies using modified antisense oligonucleotides or short interfering RNAs targeting these transcripts have been developed [121, 122]. Specifically, gapmers gave particularly interesting results. Chimeric gapmers are antisense oligonucleotides with a central continuous stretch of RNase H recruiting nucleotides (e.g., phosphorothioate DNA), flanked by nucleotides bearing affinity and stability-enhancing chemical changes, such as 2′-O-2-methoxyethyl (MOE) or LNA modifications. MOE gapmers were particularly effective and long lasting in knocking-down CUG repeats-containing RNAs in a mouse model of DM1 [123]. Interestingly, systemic administration of similarly designed gapmers was also effective for knockdown of the nuclear lncRNA Malat1 in skeletal muscle [123]. These results suggest that MOE gapmers may provide a general strategy to correct gain-of-function effects of lncRNAs and other transcripts with prolonged nuclear localization.

## 6. Conclusions

The discovery of ncRNAs as new and important regulators of gene expression has broadened our understanding of muscle biology and opened new perspectives in uncovering the mechanisms leading to muscle disease. In particular, the discovery of lncRNAs involved in muscle physiology and disease is only at the beginning and their number is certainly going to rise in the next future, offering new strategies for the development of targeted therapies.

## Figures and Tables

**Table 1:** Deregulated noncoding RNAs in skeletal muscle tissue.

Muscular dystrophy	Deregulated noncoding RNAs	References
DMD/*mdx* mice	UP: miR-21, miR-206, miR-199a-5p, miR-222, miR-31, miR-34c, miR-335, miR-379, miR-449, and miR-494	[28–36]
	DOWN: miR-22, miR-30a-3p, miR-486, miR-1, miR-29c, miR-135a, and linc-MD1	

DM1	UP: miR-206, miR-1, and miR-335	[37–40]
	DOWN: miR-29b, miR-29c, miR-33, miR-7, and miR-10	

DM2	UP: miR-221-3p, miR-34c-5p, miR-208a, miR-381, miR-34b-3p, miR-34a-5p, and miR-146b-5p	[41]
	DOWN: miR-193b-3p, miR-125b-5p, miR-378a-3p, and miR-193a-3p	

FSHD	UP: miR-1, miR-206, miR-133a, miR-133b, miR-7, miR-15a, miR-21, miR-22, miR-30e, miR-32, miR-107, miR-139, miR-152, miR-223, miR-302b, miR-331, miR-362, miR-365, miR-382, miR-496, miR-532, miR-654, and miR-660	[30, 42]
	DOWN: miR-15b, miR-20b, miR-21, miR-25, miR-100, miR-155, miR-345, and miR-594	

Only miRNAs validated either by more than one study or by one study using two independent techniques are indicated.

**Table 2:** Deregulated noncoding RNAs in serum.

Muscular dystrophy	Deregulated noncoding RNAs	References
DMD/*mdx* mice	UP: miR-1, miR-133a, miR-133b, miR-206, miR-378, miR-193b, miR-30d, miR-149, miR-30a, miR-434-3p, miR-146b, and miR-30e	[36, 43, 44]
	DOWN: miR-122, miR-429, miR-200a, miR-672, miR-31, miR-451, miR-143, miR-195, miR-148a, let-7g, miR-125b-5p, miR-200b, miR-145, miR-142-3p, let-7b, miR-26b, miR-152, let-7i, and miR-301b	

LGMD2D (*Sgca*-null mice)	UP: miR-206, miR-133a, miR-133b, miR-1, miR-378, miR-193b, miR-149, miR-30a, miR-30d, miR-709, and miR-30e	[44]
	DOWN: miR-122, miR-672, miR-125a-5p, miR-200a, miR-199a-3p, miR-195, miR-429, miR-151-3p, miR-31, miR-26a, miR-125b-5p, miR-142-3p, miR-152, miR-301b, miR-93-3p, and miR-200b	

LGMD2C (*Sgcg*-null mice)	UP: miR-133a, miR-133b, miR-206, miR-1, miR-378, miR-30d, miR-193b, miR-22, miR-149, miR-30a, and miR-106a	[44]
	DOWN: miR-125a-5p, miR-31, miR-26b, miR-142-3p, miR-429, miR-26a, miR-200a, miR-122, miR-672, miR-let-7g, miR-125b-5p, miR-let-7b, miR-let-7i, miR-215, and miR-301b	

EDMD (KI-*Lmna* mice)	UP: miR-146b and miR-200a	[44]
	DOWN: miR-130a, miR-133a, miR-133b, miR-1, miR151-3p, and miR-339-3p	

Only miRNAs validated either by more than one study or by one study using two independent techniques are indicated.
